# Tumor-derived exosomes in cancer progression and treatment failure

**DOI:** 10.18632/oncotarget.6022

**Published:** 2015-10-07

**Authors:** Shaorong Yu, Haixia Cao, Bo Shen, Jifeng Feng

**Affiliations:** ^1^ Research Center for Clinical Oncology, Nanjing Medical University Affiliated Cancer Hospital, Jiangsu Cancer Hospital and Jiangsu Institute of Cancer Research, Nanjing, Jiangsu Province, China

**Keywords:** exosomes, cancer, treatment

## Abstract

Exosomes have diameter within the range of 30-100nm and spherical to cup-shaped nanoparticles with specific surface molecular characteristics, such as CD9 and CD63. These vesicles are present in nearly all human body fluids, including blood plasma/serum, saliva, breast milk, cerebrospinal fluid, urine, semen, and particularly enriched in tumor microenvironment. Exosomes contain multiple proteins, DNA, mRNA, miRNA, long non-coding RNA, and even genetic materials of viruses/prions. These materials are biochemically and functionally distinct and can be transferred to a recipient cell where they regulate protein expression and signaling pathways. Recently, exosomes are demonstrated to have a close relationship with tumor development and metastasis. Exosomes influence therapeutic effect in cancer patients. In this review, we describe the biogenesis, composition, and function of exosomes. The mechanism on how tumor-derived exosomes contribute to cancer progression and clinical treatment failure is also described, with special focus on their potential applications in cancer therapy.

## INTRODUCTION

Most cells, including normal and diseased cells, could release bilayered membrane-bound nanovesicles into the extracellular space and body fluids. These membrane-derived vesicles (generally called extracellular vesicles) can be divided into three main classes depending on their sizes, as follows exosomes (20-100 nm), microvesicles (100-1000 nm), and apoptotic bodies (1-5 um). Apoptotic bodies are released from cells undergoing apoptosis or mechanical stress to prevent the leakage of potentially toxic cellular contents from dying cells [1]. Microvesicles are produced directly from cell plasma membrane through outward budding [2]. The term exosome is first discovered by Trams et al. in the early 1980s [3]. Exosomes are distinguished from apoptotic bodies and microvesicles by their size, origin and composition. Exosomes have diameters within the range of 30-100 nm. Exosomes have spherical to cup-shaped nanoparticles with specific surface molecular characteristics, such as CD9 and CD63. These vesicles do not originate by direct budding or shedding of plasma membrane [4]. Instead, they are formed via inward budding of endosomal membranes, thereby resulting in the formation of intracellular multivesicular bodies that later fuse with plasma membrane, and release exosomes to the exterior [5, 6]. Exosomes are present in nearly all human body fluids, including blood plasma/serum [7], saliva [8], breast milk [9], cerebrospinal fluid [10], urine [11], and semen [12]; exosomes are particularly enriched in tumor microenvironment [13, 14], thereby implying that they may play a special role in cancer development and chemoresistance. Accumulating evidence shows that tumor-derived exosomes are involved in chemoresistance. In this paper, we review some current studies investigating the effect of tumor-derived exosomes on cancer progression and the mechanism on how these exosomes contribute to cancer treatment failure.

## COMPONENT, SECRETION, ISOLATION, AND FUNCTION OF EXOSOMES

### Components of exosomes

Exosomes isolated by sequential centrifugal ultrafiltration are biochemically and functionally distinct [15]. They contain multiple proteins, DNA, mRNA, miRNA, long non-coding RNA, and even genetic materials of viruses/prions. All exosomes share common proteins or protein families, which could serve as exosome markers. These proteins include membrane-associated proteins, such as tetraspanin CD9, CD63, CD81, and CD82; cytoplasmic proteins, such as Hsp 70 and Hsp90; endosomal sorting complex required for transport-associated protein Alix and TSG101; and membrane transport and fusion proteins, such as Rab GTPases and annexins [4, 16-18]. Exosomes also contain various cell-specific proteins, depending on cellular origin and putative target function. For example, B lymphocytes and dendritic cells (DCs) secrete exosomes that carry major histocompatibility complex (MHC) class-I and class-II [19] and exosomes from activated T cells contain bioactive Fas ligand (FasL) that can induce apoptosis of T cells [20]; some tumor exosomes carry adhesion molecules, metalloproteinases, and tissue-specific proteins associated with tumorigenesis and metastasis [21-23]. Moreover, lipidomic analysis shows that exosomes also carry abundant lipid-raft cholesterol and other lipid classes [4, 24].

Besides proteins and lipids, exosomes contain large amounts of nucleic acids, such as mRNA, microRNA and long non-coding RNA and DNA. These nucleic acids could be transferred to a recipient cell by fusion of exosomes with the target cell membrane; exosomes regulate protein expression and signaling pathways in recipient cells [25]. Circulating cell-free mRNA is easily degraded by RNases in the extracellular matrix but can be protected by exosomes from degradation. RNA in exosomes is much more stable than that in plasma. The concentration of exosomal RNA has no significant differences among differential storage conditions (4, −20, −80, −20, and −20°C for 2 weeks, 2 weeks, 2 months, 3years, and 5 years, respectively) when compared with freshly prepared samples; thus, RNA is well-protected by exosomes [26]. To date, a total of 1,639 mRNA in exosomes have been reported, and this number is increasing every year. Despite the large number of RNA in exosomes, one exosome contains just only one RNA molecule or less than the average [27]. Microarray assessment of exosomes from a mast cell line confirmed the presence of approximately 1,300 different mRNA transcripts, which are approximately 8% of the mRNA detected in donor cells [25]. The exosome mRNAs are functional and can be translated after entering another cell. Some researchers found that when a mouse exosomal RNA is transferred to human mast cells, new mouse proteins could be found in the recipient cells [25].

MicroRNA (miRNA) is a class of small (19-25 nucleotides), long non-coding RNAs that play important gene regulatory roles in humans. MiRNA can be incorporated into RNA-induced silencing complex where the miRNA binds to the 3′ untranslated region of target mRNA and regulates target gene expression. MiRNAs play regulatory roles in a wide range of physiological and pathological processes. Researchers recently confirmed the existence of miRNA in exosomes: these miRNAs predominantly exist in the form of precursor miRNAs [28-31]. Although many circulating miRNAs are also reported to exist outside exosomes, some researchers demonstrated that most circulating miRNAs from serum and saliva are in exosomes [32-34]. Researchers believed that miRNAs in exosomes may be more biologically active than those outside [35]. Recent literature showed that some exosomal miRNAs are increased in serum of cancer patients, which indicated that these exosomal miRNAs might be a diagnostic marker for cancer patients [13, 36, 37]. Exosomes could also transfer miRNAs and modulate gene expression and cellular activities in recipient cells [38]. Long non-coding RNAs (lncRNAs) are another type of non-coding RNA and are usually longer than 200 nucleotides. These RNAs regulate the expression of associated genes at transcriptional, posttranscriptional, and epigenetic levels [39]. Recently, lncRNAs are found to exist in exosomes and could function as potential stable biomarkers for cancer patients [40, 41]. Although not all exosomes contain DNA, some tumor cell-derived exosomes did contain double-stranded DNA (dsDNA) [42], and these DNAs are generally larger than 2.5 kb. These DNAs span all chromosomes with KRAS and p53 mutations in serum exosomes of pancreatic cancer patients [43, 44].

Exosomes also contain genetic materials of viruses/prions as well. Epstein-Barr virus (EBV)-infected nasopharyngeal carcinoma cells release exosomes that carry the EBV-encoded latent protein 1 (LMP1), mature micro-RNAs (EBV-miRNAs), and EBV-encoded latent phase mRNAs [45-49]. Exosomes secreted from hepatitis C virus-infected cells contain full-length viral RNA and protein. These exosomes can transmit infection to other hepatoma cells and establish a productive infection [50, 51]. Bukong TN et al. showed that exosomes isolated from the sera of chronic hepatitis C virus (HCV)-infected patients contain HCV RNA: these exosomes could mediate viral receptor-independent transmission of HCV to hepatocytes [52].

Exosome components come from the original cells; theoretically, these components could partly reflect the content of the original cells. However, whether or not these exosomes could dependably represent the components of original cells is still up for debate. A study from Hong BS showed that most of the mRNA transcripts from SW480 cells exist in SW480-derived microvesicles, which implies the similarity in components of exosomes and donor cells [53]. Another study demonstrated that exosomal RNA content of human breast cancer cell line could reflect the RNA content of the donor cells [54]. A similar study also revealed that the molecular profile of hypoxic exosomes could reflect the hypoxic response of malignant brain tumor glioblastoma multiforme (GBM) donor cells and GBM patient tumors [55]. More importantly, a strong correlation of mRNA and miRNA signals between tumor cells and exosomes was observed [56, 57]. Although many studies showed that cells’ exosomes are similar to the profile of the corresponding parent cells [57, 58], other studies also proved that the protein and RNA contents of exosomes are different from those of the original cells [59-62]. Valadi H et al. showed the presence of approximately 1,300 mRNA in exosomes, many of which are absent in the cytoplasm of the donor cell. These identified mRNAs in exosomes are only approximately 8% of the mRNA detected in the donor cells [25], thereby indicating that exosomal gene expression profile could not reflect the profile of the donor cells. Exosome secretion is an energy-requiring process and could be modulated by extracellular signals to perform a specific task. Thus, realistically reflecting the profile of the donor cell is difficult.

### Secretion modulation and isolation of exosomes

The molecular mechanisms of exosome biogenesis and secretion are poorly understood; however, the release of exosomes is precisely regulated by multiple signal molecules [63]. Generally, exosome release is affected by multiple factors, as follows: ceramide synthesis, calcium signaling, p53, acidosis, and heat and cellular stresses [64-71]. Exosome secretion is mainly controlled by conserved families of cytosolic proteins, such as the Rab family of small GTPases. Rab proteins, especially Rab27a and Rab27b, can control the different steps of the exosomes secretion pathway [72-74]. In addition to Rab27a and Rab27b, proteins belonging to another Rab family (Rab11 and Rab7) promote docking and fusion of exosomes in a calcium-dependent manner [73, 75]. Rab 35 can regulate exosome secretion by interacting with GTPase-activating protein TBC1 domain family, member 10A-C (TBC1D10A-C) [35, 76]. Furthermore, exosome biogenesis and release are modulated by PARK9, Vps4A, and lysosome-related organelle-associated ATP-binding cassette transporter A3 [77-79].

The most accepted method for exosome isolation is based on ultracentrifugation (UC) [80]. This method can obtain minimally contaminated pellets of exosomes; however, it demands a complicated and prolonged process. Later, System Biosciences (www.systembio.com) developed a proprietary reagent named ExoQuick (EQ) that can precipitate exosomes when added to several types of biological fluids. EQ has proved it efficiency in several experimental settings [81, 82]. In 2009, Logozzi M et al designed an in-house sandwich ELISA (Exotest) to capture and quantify exosomes in plasma based on the expression of housekeeping proteins (CD63 and Rab5b) and a tumor-associated marker (caveolin-1) [83]. Using this immunocapture-based test, they found that plasma from cancer patients has significantly higher levels of exosomes than that from healthy donors. A similar method was also developed to capture CD43+ blast-derived exosomes in acute myeloid leukemia [84]. To improve the accuracy of the method, an extracellular vesicle array is established to phenotype exosomes directly from the plasma samples [85].

### Function of exosomes

Initially, exosomes are viewed as “garbage bags” that allow cells to export waste products, molecules no longer as useful as cells differentiate, and molecules, such as drug that may be harmful to cells [86]. Exosomes are important conveyors of immune response and present antigen to the antigen-presenting cell (APC) [87]. Subsequently, many cell types release exosomes *in vitro*, and the function of exosomes has been intensively studied. Although the detailed function of exosomes in physiological and pathological regulation is still being studied, the main function of exosomes is to participate in cell-to-cell communication by transferring bioactive molecules to recipient cells close to or distant from the original cells; exosomes subsequently alter the content and behavior of the recipient cell [4, 88]. This may result from direct membrane fusion or endocytosis of exosomes into target cells.

Exosomes may be a natural shuttle for the somatic-to-germline transmission of RNA. Cossetti C and their colleagues generated a mouse model xenografted with human melanoma cells stably expressing EGFR-encoding plasmid and found that EGFR RNA was released from the xenografted human cells into the bloodstream and eventually in spermatozoa of mice [89]. This result proved that exosomes are carriers of an information flow from somatic cells to gametes.

**Figure 1:**
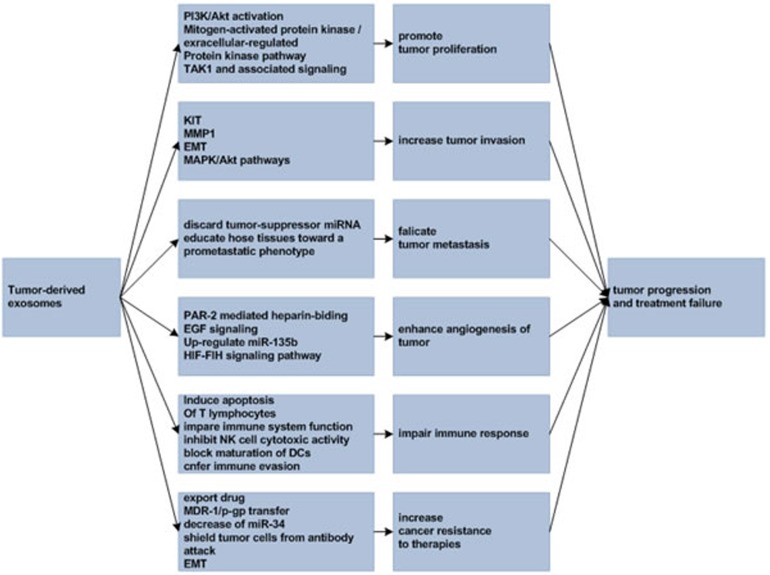
Tumor-derived exosomes in tumor progression and treatment failure

Exosomes are closely related to tumor development and metastasis and influence therapeutic effect in cancer patients. As an example, triple negative breast cancer cells are more aggressive and are inherently resistant to multiple anticancer drugs. Research from O'Brien K et al found that exosomes from triple negative breast cancer cells can transfer phenotypic traits representing their cells of origin to secondary cells and confer increased invasiveness to other cells [90]. In the following sections, we will discuss the involvement of exosomes in cancer development and treatment failure (Figure 1).

## TUMOR-DERIVED EXOSOMES IN CANCER DEVELOPMENT AND TREATMENT FAILURE

### Tumor-derived exosomes promote progression, invasion, and metastasis of cancer cells

Invasion is the main characteristic of cancer, and most cancer treatment failures are attributed to metastasis because metastatic lesions are generally refractory to multiple forms of available therapies. Metastasis is a multistep process that involves the acquisition of migratory malignant phenotype; this process allows cancer cells to invade normal human organs, and disseminates and establishes new growth at distant sites [91]. Tumor derived-exosomes can provide autocrine, paracrine, endocrine, and other signals that can facilitate cancer growth, maintain its invasion, and promote metastasis.

Hong BS et al. found that colorectal cancer cell-derived exosomes are enriched in cell-cycle-related mRNAs and can promote proliferation of endothelial cells, thereby suggesting that exosomes of cancer cells could be involved in tumor growth and metastasis [53]. Another study by Qu JL showed that gastric cancer cell line SGC7901-derived exosomes could promote proliferation of both SGC7901 cells and other gastric cancer cell line BGC823 cells. This promotion is partly achieved by the activation of PI3K/Akt and mitogen-activated protein kinase/extracellular-regulated protein kinase pathways [92]. A similar result was obtained in hepatocellular carcinoma cells whose exosomes could enhance hepatocellular cancer cell growth by modulating transforming growth factor β activated kinase-1 expression and associated signaling [59]. In addition to tumor cells, exosomes of other cells (i.e. macrophages, human bone marrow mesenchymal stem cells, and mast cells) could also promote cancer cell proliferation by different signaling pathways [93-95].

During tumor invasion, constant communication occurs between tumor cells and surrounding stromal cells via exosomes. Gastrointestinal stromal tumor cells invade the interstitial stroma by releasing oncogenic protein tyrosine kinase-containing exosomes, thereby triggering the phenotypic conversion of progenitor smooth muscle cells to tumor-promoting cells [96]. This is accomplished through stimulation of matrix metalloproteinases 1 (MMP1) production by stromal cells via uptake of tumor-derived exosomes. Treatment of EBV-negative cells with EBV-positive cells derived exosomes could increase migration and invasiveness of nasopharyngeal cell lines in functional assays, which might correlate with the phenotype associated with epithelial-mesenchymal transition (EMT) [97]. Evidence shows that tumor cells can share some malignant characteristics. For example, exosomes isolated from mutant KRAS-expressing colon cancer cells contain many tumor-promoting proteins, including KRAS, EGFR, SRC family kinases, and integrins; they can transfer their invasiveness to recipient cells that express wild-type KRAS gene [98]. Another evidence also found that although only a small percentage of glioma cells can express EGFRvIII (a truncated and oncogenic form of the epidermal growth factor receptor), most of the cells lacing EGFRvIII within the tumor can exhibit a transformed phenotype via exosomes, effectively activate transforming signaling pathways (MAPK and Akt), and increase tumor cell growth capacity and invasiveness [99]. Similar effects have also been observed in breast cancer cells [100].

Metastasis is a complex multistep process and is a hallmark of malignant tumor. To metastasize, cancer cells must manipulate the microenvironment and optimize conditions for migration and implantation both locally and at distant metastatic sites [101]. This process requires a complex tumor-stromal interaction that involves various intercellular communication [91]. Exosomes play vital roles in cancer metastasis. They could discard tumor suppressor miRNA, such as miR23b, to acquire metastatic properties and facilitate cancer cell metastasis [74]. Exosomes released by melanoma cells can prepare sentinel lymph nodes for tumor metastasis by enhancing migration of melanoma cells to melanoma exosome-rich sites in sentinel lymph nodes [102]. Furthermore, tumor exosomes can educate selected host tissues toward a prometastatic phenotype and create a permissive environment at potential metastatic sites [73, 103-106]. Although these phenomena are observed in different tumor models, research on the precise underlying mechanisms is still needed.

### Tumor-derived exosomes enhance angiogenesis of cancer cells

Hypoxia is another important feature of solid tumor especially advanced disease due to an imbalance in the supply and consumption of oxygen by tumor cells [107]. Hypoxic tumor exhibits more aggressive phenotypes. Tumor cells under hypoxia can produce a secretion partly in the form of exosome that modulates the microenvironment to facilitate tumor angiogenesis and metastasis. Moreover, tumor angiogenesis is largely mediated by the hypoxia-inducible factor (HIF) family of transcription factors [107-109].

Hypoxia could promote the release of exosomes in cancer cells; this hypoxic response might be mediated by the HIF oxygen sensing pathway [110, 111]. When breast cancer cells are exposed to modest (1%) and severe (0.1%) hypoxia, the amount of exosome-size nanoparticles harvested from the conditioned media can increase by 32.3±4.8% and 90.9±7.1% respectively [110]. This exosome increase is suggested to modulate tumor cell microenvironment and enhance angiogenic potential of tumor tissues by protease-activated receptor 2-mediated heparin-binding EGF signaling in endothelial cells [64, 112]. Exosomes could also reflect the hypoxic status of glioma cells. Exosomes derived from brain tumor glioblastoma multiforme cells grown at hypoxic condition are potent inducers of angiogenesis both ex vivo and *in vivo* via phenotypic modulation of endothelial cells [55]. In a multiple myeloma (MM) model, hypoxia-resistant MM cells produced more exosomes that contain significantly upregulated miR-135b; this exosomal miR-135b could enhance endothelial tube formation under hypoxia via HIF-FIH signaling pathway [111].

Exosomes enhance angiogenesis to alleviate the hypoxia state of cancer; however, these newly formed blood vessels under hypoxia are chaotic and disordered. Normalization of vasculature may improve the delivery of chemotherapeutics and tumor sensitivity to radiation [113, 114]. Therefore, inhibition of exosome secretion or removal of cancer cell-derived exosomes could be a promising method to inhibit or normalize the angiogenesis of tumor cells. In clinical practice, inhibition of angiogenesis can be achieved via administration of a number of molecularly targeted agents such as bevacizumab. The addition of bevacizumab to chemotherapy can prolong survival of patients with metastatic colorectal cancer and recurrent or advanced non-small cell lung cancer [115, 116]. The US Food and Drug Administration has approved the use of bevacizumab in metastatic colorectal cancer and recurrent or advanced non-small cell lung cancer, which suggests and encourages the switching off of tumor exosomes’ promotion on angiogenesis, thereby improving the outcome of cancer treatment.

### Tumor-derived exosomes suppress immune responses and assist cancer progression

During cancer development and metastasis, most cancer cells are opposed by the human immune system. Suppressing immunoreaction or escaping from immune surveillance is a vital task for cancer development. In this process, exosomes produced by both immune and non-immune cells play important roles in the regulation of host immunity. Most cancer patients are in a state of immunosuppression or immunodeficiency. Recently, immunotherapy plays important roles in many cancers, such as malignant melanoma, renal cancer, liver cancer, and even non-small cell lung cancer [117-119]. In this section, we will summarize and discuss how exosomes modulate cancer patients’ immunoactivity to assist in cancer cells development.

In the past decades, researchers found that extracellular vesicles released by B cell lines contain MHC class II, which can directly stimulate CD4+T cell clones [120]. Later study proved that the vaccination of mice with exosomes derived from tumor peptide-pulsed DCs can stimulate an anticancer immune response and suppress tumor growth in a T cell-dependent manner [121]. Vaccination of mice with tumor-derived exosomes can also induce CD8+ T cell-mediated antitumor effect on autologous tumors and other tumors [122]. Tumor-derived exosomes that contain cancer cell antigen could be used as a source of tumor antigen to stimulate an antitumor response. However, antigen presentation is only one of the functions of exosomes. Despite that some studies have shown that tumor-derived exosomes can activate immune system [4, 123-125], substantial evidence suggests that tumor-derived exosomes actually suppress antigen-specific and nonspecific antitumor responses.

Although tumor exosomes express tumor antigen that leads to their proposed use as tumor vaccines, these exosomes also confer antigen-specific immunosuppression [126]. A large body of evidence points to the established role of tumor-derived exosomes in facilitating immunosuppression [127, 128]. For example, exosomes from OVA-expressing melanoma, which contained full-length OVA protein, could suppress an OVA-specific immune response effectively [129]. In a tumor-bearing model, plasma-derived exosomes, which were positive for CD11b, suppressed tumor antigen-specific response through a MHC class II-dependent mechanism [130]. The detailed mechanisms of exosomes in suppressing tumor antigen-specific response have been intensively studied. The following paragraphs may partly provide some potential explanations.

Fas (CD95), a type I transmembrane glycoprotein, and FasL, a type II transmembrane protein, both belong to the tumor necrosis factor superfamily of receptors. The Fas/FasL system is an important mediator of apoptosis in the immune system. Co-expression of Fas and FasL by activated T cells usually leads to activation-induced cell death [131]. FasL is generally expressed by activated T lymphocyte, a natural killer (NK) cells [132, 133]. FasL expression is also found in many tumors, such as melanoma, lung, and ovarian carcinoma; Fas/FasL system plays an important role in controlling survival and growth of tumor cells [134, 135]. However, most tumors are resistant to apoptosis induced by Fas signaling by synthesizing a protective protein [136]. Conversely, exosomes derived from both tumor and activated human T cells containing bioactive FasL can induce apoptosis of T lymphocytes and impair immune system function of cancer patients [126, 137-141]. Moreover, exosomes expressing Fas L from activated CD8+ T cells of mice can promote melanoma, lung, and nasopharyngeal cancer cell invasion and metastasis by increasing the expression of matrix metalloproteinase (MMP) 9 [20, 142]. Cancer exosomes can suppress T cells’ function by both Fas/FasL signaling pathway and adenosine production, thereby transforming growth factor-β [143, 144]. Exosomes derived from the ascites of ovarian cancer patients express death ligands, FasL, and TRAIL [145]. These death ligands can partly account for in the immune system, thus inhibiting a tumor growth inhibitory immune response.

Interleukin-2 (IL-2) is important in the homeostasis of lymphoid cells and supports the expansion and differentiation of CTL and NK cells. However, tumor-derived exosomes can impair the proliferative response to IL-2 in all lymphocyte populations; this effect is mediated principally by the CD4+ T-cell subset [146]. Tumor exosomes caused immunosupression not only on T cells but also on NK cells. Experimental evidence found that exosomes produced by TS/A or 4T.1 murine mammary tumor cells resulted in accelerated growth of implanted tumor cells in syngeneic BALB/c and nude mice [147]. Furthermore, this effect was achieved by inhibiting NK cell cytotoxic activity and IL-2-stimulated NK cell proliferation [147]. Tumor-derived exosomes can also block the maturation of DCs and macrophages both *in vivo* and *in vitro* through a TGFβ1-dependent mechanism [148].

Tumor-derived exosomes not only inhibit immune activity of multiple lymphoid cell types but also confer immune evasion by down-modulating NKG2D expression in cancer [149, 150]. NKG2D is an activating receptor for NK, NKT, and CD8+ T cells, and its down-regulation of expression or loss in cancer cells is a key mechanism underlying immune evasion. An important study from Clayton A showed that exosomes produced by various cancer cell lines *in vitro* or isolated from pleural effusions of mesothelioma patients carried NKG2D ligands. These exosomes can trigger down-regulation of surface NKG2D expression in NK cells and CD8+ T cells. Thus, lymphocyte activation through NKG2D following exosome treatment is impaired [151]. Furthermore, NKG2D ligand-positive tumor-exosome interaction with lymphocytes did not lead to the activation of CD8+ T or NK cells. NKG2D down-modulation was due to direct exosomal delivery of TGFβ1 to CD8+ T or NK cell subset [151].

### Tumor-derived exosomes increase cancer cell resistance to therapies

Traditionally, exosomes are in charge of waste product export and less needed molecules from cells. In cancer cell models, exosomes could also export chemotherapeutic drugs, which partly play a role in cancer cell resistance to chemotherapy. For example, when factors associated with exosomes shedding are combined into a vesicle-shedding index, the vesicle shedding index of the NCI60 cell line panel is positively correlated with the compounds’ sensitivity to cancer cells for most of the 171 compounds of the National Cancer Institute Standard Anticancer Agent Database: this observation supports the hypothesis that exosome shedding and drug resistance are related [35, 152]. Particularly, encapsulation and expulsion of doxorubincin in vesicles shedding into the surrounding medium have been observed. Relative differences in the rate of vesicle shedding corresponded well with doxorubicin resistance across various cell lines [152]. However, not all cases of anticancer drug resistance are associated with the shedding of exosomes, with the exception of 5-Fu and staurosporine. Both 5-Fu and staurosporine are cell permeable and show little affinity for shed vesicle/exosomes. Concurring with this finding, other authors observed that exosomes released from cisplatin (DDP)-resistant human ovarian cancer cells contained 2.6-fold more DDP than those released from sensitive cells. The enhanced exosomal export was accompanied by higher exosomal levels of putative DDP export transporters MRP2, ATP7A, and ATP7B [153]. Exosome-mediated drug expulsion is now an emerging concept in the area of drug resistance.

Drug resistance is a multifaceted problem, and exporting drug by exosomes is only a part of the underlying mechanism. Exosomes contribute to chemoresistance to cancer cells in multiple ways. When DDP is added to lung cancer cells (A549), exosome secretion is strengthened, and the addition of this secreted exosomes to other A549 cells can increase the resistance of these cells to DDP [68]. When A549 is exposed to DDP, the expression levels of several miRNA and mRNA, which are reportedly associated with DDP sensitivity, change significantly in secreted exosomes. This phenomenon implies that the changes of potential associated miRNA and mRNA may mediate the resistance of A549 cells to DDP, but the precise underlying mechanisms are still being studied. In prostate and breast cancer models, the same phenomenon is also observed: exosomes expelled from docetaxel-resistant cancer cells confer docetaxel-resistant to docetaxel-sensitive cancer cells, which may be partly due to exosomal MDR-1/p-gp transfer [154, 155]. Subsequently, the decrease of miR-34 in docetaxel-resistant prostate cancer cells and cell-derived exosomes is a response to docetaxel resistance [57]. However, authors did not explain how this decrease of miR-34 in exosomes is involved in the resistance of other prostate cancer cells to docetaxel. In a hepatocellular cancer (HCC) model, a long non-coding RNA ROR enriched in exosomes from HCC cells can reduce chemotherapy-induced cell death in recipient cells by mediating TGFβ-dependent chemoresistance [41]. Inhibitors of apoptosis (IAP) can regulate cell survival and are often deregulated in cancers. The high levels of IAP expression in cancer cells are associated with disease progression and therapy resistance [156, 157]. Exosomes secreted from human cancer cell lines contain full-length IAP mRNA transcripts, which may be translated into functional proteins upon reabsorption into recipient cells and may increase cell resistance to anticancer drugs [158, 159]. Exosome-mediated transfer of miRNAs within the tumor microenvironment increases neuroblastoma's resistance to chemotherapy [160]. The use of an exosome inhibitor (GW4869) significantly restores neuroblastoma cell sensitivity to CDDP statistically, thereby providing the possibility of using exosome inhibitors to prevent or overcome drug resistance. In addition to tumor-derived exosomes, tumor-associated fibroblasts-derived exosomes also contribute to chemoresistance of colorectal cancer by priming cancer stem cells [161].

Beyond traditional chemotherapy drugs, monoclonal antibody-based therapy has evolved as a mainstay of targeted anticancer therapy and has allowed both direct and immune-mediated cell lysis. Anti-CD20 chimeric antibody rituximab has significantly improved the overall survival of malignant B-cell lymphoma and becomes the standard of immunotherapy in malignant B-cell lymphoma to date [162, 163]. Although high response to rituximab is observed in malignant B-cell lymphoma, exosomes derived from lymphoma could still impede its activity or even confer resistance to rituximab. B-cell lymphoma cells release exosomes carrying CD20. These exosomes can bind therapeutic anti-CD20 antibodies, consume complement, and protect target cells from rituximab attack [77]. Approximately half of all the plasma rituximab was fixed to exosomes 3 h after rituximab infusion. Trastuzumab is another monoclonal antibody that can target human epidermal growth factor receptor 2 (HER2) receptor on breast cancer cells and is widely used in the treatment of breast cancer patients [164]. Exosomes released by HER2-overexpressing breast cancer cell lines express a full-length HER2 molecule. These exosomes secreted either from HER2-positive tumor cell-conditioned supernatants or from breast cancer patients’ serum can bind to trastuzumab, can inhibit binding of trastuzumab to tumor cells, and can reduce antibody-dependent cellular cytotoxicity of HER2-overexpressing breast cancer cells [165, 166]. Results of these studies strongly indicate that exosomes generated from tumor cells contain surface molecules or antigen of the original cells and can efficiently shield tumor cells from antibody attack, and can result in failure of the target treatment in cancer patients.

EMT is driven by a complex interaction network and is one of the hallmarks of cancer. In the EMT process, epithelial cells undergo a shift in plasticity and acquire the ability to disseminate, metastasize and resist drugs. Cancer cells that underwent EMT are usually resistant to multiple anticancer drugs, and this transition could help cancer cells in escaping from environmental stress [167]. EMT inducers, such as MMP, IL-6, TGFβ, annexin A2, integrin 3, and hepatoma-derived growth factor, have been found in some tumor cell-derived exosomes, thereby suggesting that cancer cell-derived exosomes might participate in the EMT process in cancer cells [168-172]. Among these inducers, the WNT signaling pathway is a well-studied pathway that can promote gene expression program and can favor EMT [173]. Human-derived exosomes contain WNT, and can be transferred to recipient cells, and subsequently activate WNT signaling pathway; thus, exosomes may have a close relationship with the EMT of cancer cells [174-176]. A recent study found that exosomes generated from nasopharyngeal carcinoma (NPC) contain LMP1, a principal oncoprotein of EBV that can drive oncogenic process and tumor progression of NPC [97]. Authors showed that EBV-negative cell lines treatment with LMP1 exosomes increases migration and invasiveness of NP cell lines, which correlates with the phenotype associated with EMT [97]. Meanwhile, another important study from Josson S et al. proved that stromal fibroblast-derived exosomes that carry abundant miR-409 can induce EMT of the adjacent epithelia in the tumor compartment of prostate cancer [177]. Despite that the number of studies on this topic is limited, a preliminary conclusion can be drawn as follows: exosomes derived from tumor cells are associated with EMT, and such association might consequently influence the chemosensitivity of anticancer drugs [178].

Cancer cells’ intrinsic resistance to cytotoxic drugs is a main issue in cancer therapy. Microenvironmental acidity is a simple but highly efficient mechanism of chemoresistance. A key factor in this resistance is the “reversed pH” gradient [179, 180]. A low pH condition is a hallmark of tumor malignancy, and many drugs are weak bases. Under normal conditions (neutral outside and weakly acidic inside), drugs are attracted within cells. However, when entering the acidic tumor microenvironment, these drugs are quickly protonated and therefore neutralized. In an acidic setting, an increased exosome release and uptake cab be observed [110, 181]. The plasmatic exosome levels are correlated with tumor size [83]. Low pH values select more aggressive acid-resistant clones and favor tumor invasion and chemoresistance; the increased exosomes secreted by tumor cells are probably involved in this process. Moreover, tumor acidity negatively regulates tumor-specific effector T cells and contributes to immune suppression [182, 183]. Tumor-derived exosomes suppress immune responses. Conceivably, tumor acidity suppresses immune response via increased tumor-derived exosome release. Recently, several studies showed that proton pump inhibitors (PPIs) can be useful in modulating tumor acidification and overcoming the acid-related chemoresistance both *in vitro* and *in vivo* [179, 184-186]. These findings are also supported by clinical studies in both animals and patients with osteosarcoma [187-189]. Pretreatment with PPIs also leads to the inhibition of exosome uptake by tumor cells [181]. This observation implies that PPIs increase tumor cells’ chemosensitivity to anticancer drug probably via inhibition of exosome secretion of tumor cells.

### Applications of exosomes to cancer therapy

The main function of exosomes is to deliver various biomolecules, including proteins, peptide ligands, DNA, and RNAs. When exosomes are designed or selected to contain specific bioactive molecules, they could be used to deliver anti-tumor molecules or drugs to treat tumors. In other research areas, exosomes are designed to deliver anti-inflammatory agents, such as curcumin, to enhance anti-inflammatory activity [190, 191]. Some studies in cancer treatment are also available. For example, phosphatase and tensin homolog deleted on chromosome 10 (PTEN), a tumor suppressor protein, was found to exist in exosomes from mouse embryonic fibroblasts and human embryonic kidney cells. PTEN can be internalized by recipient cells and reduce cellular proliferation [192]. Genetically engineered microvesicles/exosomes carrying suicide mRNA/protein can lead to tumor regression upon systemic treatment with prodrug (5-Fu) [193]. Accordingly, another study showed that designed exosomes can efficiently deliver let-7a miRNA to EGFR-expressing breast cancer cells [194]. Targeting is achieved by engineering the donor cells to express the transmembrane domain of platelet-derived growth factor receptor fused to the GE11 peptide which can bind specifically to EGFR [194]. However, all of applications are in the experimental stage. Further study is needed to realize the usefulness of exosomes in clinical practice because many critical challenges must be overcome.

Another application of exosomes for therapeutic development is their use in tumor vaccination; tumor-derived exosomes commonly contain many tumor antigens for APC activation, including dendritic cells, which consequently induce cytotoxic T lymphocyte-dependent antitumor response [122, 195]. Encouraged by these discoveries, several clinical trials are completed in the 2000s. The first phase I clinical trial reported by Escudier B showed that when 15 metastatic melanoma patients were treated with a vaccination of autologous dendritic cell (DC)-derived exosomes, no grade II toxicity and maximal tolerated dose was achieved; thus, the safety of exosomes administration in indicated [196]. Moreover, treatment efficacy was found in four patients, with one minor, two stable, and one mixed responses [196]. However, in this clinical trial, authors failed to detect the vaccine-specific T-cell response while observing potent dendritic cell-derived exosome (Dex)-related NK cell activation [197]. Authors found that Dex vaccines can significantly augment circulating NK cell numbers and NKG2D-dependent functions in 7/14 patients [150].

Another similar phase I clinical study is the treatment of non-small cell lung cancer patients with autologous DC-derived exosomes loaded with MAGE tumor antigens [198]. Concurring with the former study, this clinical trial proved that production of exosome vaccine is feasible, and exosome therapy is well-tolerated in patients with advanced NSCLC because only grade 1-2 adverse events related to the use of exosomes are observed [198]. Although Dex is intended to activate antigen specific, MHC-restricted T cell response, researchers found minimal increase in antigen-specific T cell activity in vitro assays, whereas increase in NK activity following immunization in 2/4 patients is observed. Researchers speculated that cytokines released in response to Dex therapy could cause NK cells activation, or Dex could directly activate NK cells. Subsequently, in 2008, results from a phase I clinical study in China were reported by Dai S. Ascite-derived exosomes combined with the granulocyte-macrophage colony-stimulating factor (GM-CSF) or exosomes alone are used to treat a total of 40 colorectal cancer patients. Results showed that both therapies are safe and well-tolerated. Exosomes plus GM-CSF, but not exosomes alone, can induce beneficial tumor-specific antitumor cytotoxic T lymphocyte response [199].

Despite the safety and tolerance of exosome vaccines, phase I clinical trials failed to show the immunizing capacity of these vaccines. These low immunogenic capacities promoted researchers to develop second-generation exosome vaccines with enhanced immunostimulatory properties [197, 200]. Data revealed that metronomic cyclophosphamide can facilitate Dex-mediated T cell priming and restore T and NK cell functions in end-stage patients [201, 202]. Table 1 lists clinical trials on exosomes and cancer in www.clinicaltrials.gov. In some of these trials, exosomes were used as cancer vaccines, biomarkers, and drug delivery media.

**Table 1:** Clinical trials concerning exosomes and tumor

Exosome type	Cancer type	Phase	status	notes	Ref.
Plant exosomes	Colon cancer	I	recruiting	Drug delivery	NCT01294072
Patient exosomes	Metastatic melanoma	-	Not yet recruiting	Molecular mechanism study	NCT02310451
Patients exosomes	Gastric cancer	Case control	recruiting	Biomarker study	NCT01779583
cell exosomes	Oropharyngeal cancer	Case control	Not yet recruiting	Biomarker study	NCT02147418
Edible plant exosome	Head and neck cancer	I	recruiting	Treatment study	NCT01668849
Dendritic cell exosome	Unresectable non-small cell lung cancer	II	unknown	Treatment study	NCT01159288
Patients exosomes	Malignant glioma of brain	I	completed	Treatment study	NCT01550523

Although tumor exosomes often contain tumor-specific antigens, they carry more tumor-activating molecules. These molecules enhance tumor proliferation, metastasis, confer immunosupression of host, and participate in cancer therapy resistance. Thus, strategy for specifically targeting exosomes is a promising therapeutic option for cancer treatment. Some researchers found that exosome depletion from the plasma of aggressive B-cell lymphoma patients can increase cytolytic efficacy against cell line targets. The cytolytic activity of rituximab-containing plasma after infusion against the patient's autologous tumor cells is also significantly enhanced by exosome depletion [77]. In 1989, Lentz MR found that simply using UltraPheresis procedure, six of the 16 metastatic cancer patients received about 50% or more tumor reduction [203]. Recently, Aethlon Medical devised a therapeutic hemofiltration approach, termed as Aethlon ADAPT^TM^ (adaptive dialysis-like affinity platform technology) system [204]. This system can capture innumerable antibodies and other affinity reagents, such as aptamers, proteins ligands, and exosomes. Hemopurifier^®^, the first ADAPT^TM^ device, reduced viral load in HCV-infected patients who are not concurrently receiving anti-viral drugs [205]. A clinical safety study of the Aethlon Hemopurifier^®^ in chronic end-stage renal disease patients with HCV infection is underway (NCT02215902). However, whether this device will obtain a positive result in cancer patients is still unknown and needs further validation. In addition to blood purification, PPIs can improve low pH conditions of cancer cells; PPI pre-treatment *in vivo* also induces a clear reduction in the plasmatic levels of tumor-derived exosomes, thereby suggesting that PPI might be a good choice for secretion inhibition of tumor-derived exosomes [206].

## SUMMARY

Communication among tumor cells and between tumor cells and human organs is crucial for cancer progression. Exosomes are emerging as a major player in this communication, specifically in cancer development and progression. Tumor-derived exosomes contribute to the failure of cancer treatment, and eliminating these exosomes seems suitable for cancer therapy. As more studies focus on the important roles of exosomes in cancer development, research on exosomes is vital for understanding cancer. Despite that many studies have been published on this topic, the means by which exosomes assist in cancer development and confer cancer treatment failure are still unclear. We published the data and phenomena that we observed, but detailed underlying mechanisms still need deeper investigation.
